# Multivariate regression analyses of data from a randomised, double-blind, placebo-controlled study confirm quality of life benefit of epoetin alfa in patients receiving non-platinum chemotherapy

**DOI:** 10.1038/sj.bjc.6600657

**Published:** 2002-11-26

**Authors:** L Fallowfield, D Gagnon, M Zagari, D Cella, B Bresnahan, T J Littlewood, P McNulty, G Gorzegno, M Freund

**Affiliations:** Cancer Research UK Psychosocial Oncology Group, Brighton and Sussex Medical School, University of Sussex, Brighton, BN1 9QG, UK; Johnson & Johnson Pharmaceutical Research & Development, L.L.C., 700 Route 202, Raritan, New Jersey, NJ 08869, USA; Ortho Biotech UKI, PO Box 829, Saunderton High Wycombe, Bucks, HP14 4HJ, UK; Institute for Health Services Research and Policy Studies, Northwestern University, 1001 University Place, Evanston, Illinois, IL 60201, USA; John Radcliffe Hospital, Headington, Oxford, OX3 9DU, UK; Ospedale S. Luigi, Orbassano, Via Regione Gonzole 10, Orbassano, 10126 Italy; University of Rostock, Postsach 100888, Rostock, 18055 Germany

**Keywords:** anaemia, cancer, epoetin alfa, haemoglobin, non-platinum chemotherapy, quality of life

## Abstract

Cancer-related anaemia is associated with a wide spectrum of symptoms that can negatively affect quality of life. Because epoetin alfa has demonstrated efficacy in correcting cancer-related anaemia, the impact of this treatment on quality of life was evaluated in a multinational, randomised, double-blind, placebo-controlled trial in 375 anaemic cancer patients receiving non-platinum-based chemotherapy. The cancer-specific measures of quality of life included the general scale (FACT-G Total) and fatigue subscale (FACT-An Fatigue subscale) of the Functional Assessment of Cancer Therapy-Anaemia and the Cancer Linear Analogue Scales measuring energy, ability to do daily activities, and overall quality of life. These measures were also used to examine the relationship between haemoglobin levels and quality of life. Both univariate and multiple linear regression analyses of quality of life data were performed. Results of the univariate analysis have been reported previously. The *a priori-*planned multiple linear regression analysis, which accounted for the effects of disease progression and several other possibly confounding variables on quality of life, showed a significant advantage for epoetin alfa over placebo for the five scales (all, *P*<0.05), and confirmed the results of the univariate analysis. For cancer-specific measures, significant correlations were demonstrated between baseline haemoglobin and quality of life (*r*, range: 0.14–0.26, all *P*<0.05) and between change in haemoglobin and change in quality of life (*r*, range: 0.26–0.34, all *P*<0.01). These findings provide evidence that increasing haemoglobin levels by epoetin alfa administration can significantly improve cancer patients' quality of life.

*British Journal of Cancer* (2002) **87**, 1341–1353. doi:10.1038/sj.bjc.6600657 www.bjcancer.com

© 2002 Cancer Research UK

## 

Fatigue has replaced pain, nausea, and vomiting as the most common and often the most troubling side effect of concern to cancer patients receiving chemotherapy, with cancer patient surveys in both Europe and the USA reporting prevalence rates between 58% and 90% (Vogelzang *et al*, 1997; Stone *et al*, 1998, 2000; Simon and Zittoun, 1999; Curt *et al*, 2000). In addition, in a recent survey of 379 individuals who had completed chemotherapy, 37% reported at least 2 weeks of fatigue in the previous month. Of respondents who had completed treatment over 5 years ago, 33% still reported at least 2 weeks of fatigue in the month before the interview (Cella *et al*, 2001). The aetiology of fatigue in cancer patients is multifactorial and may include adverse effects of treatment, underlying disease, metabolic and physiological abnormalities, and other variables (Groopman, 1998). Although fatigue can have a deleterious effect on the quality of cancer patients' lives, it remains poorly understood, under-investigated, and often under-treated (Stone *et al*, 2000). Anaemia occurs frequently in cancer patients (Barrett-Lee *et al*, 2000; Coiffier *et al*, 2001) and is highly associated with fatigue and diminished quality of life (QoL) (Cella, 1998).

Cancer-related anaemia may be a consequence of the neoplastic process itself; cytotoxic therapy; marrow infiltration by malignant cells; renal impairment; iron, folate, or vitamin B_12_ deficiency; infectious processes; or blood loss (Ludwig and Fritz, 1998). Anaemia can give rise to exhaustion, fatigue, dizziness, headache, dyspnoea, chest pain, and impaired cognitive function, all of which can diminish patients' QoL (Winningham *et al*, 1994; Cella, 1998; Ludwig and Fritz, 1998; Schwartz, 1998). Many cancer patients require therapeutic intervention to correct the underlying anaemia. In several studies reported between 1990 and 2001, recombinant human erythropoietin (r-HuEPO, epoetin alfa) increased haemoglobin (Hb) levels and subsequently ameliorated anaemia in cancer patients. Moreover, in the studies that evaluated QoL, epoetin alfa provided benefits ranging from improvement in energy and activity levels to an overall improvement in QoL (Abels, 1992; Leitgeb *et al*, 1994; Glaspy *et al*, 1997; Demetri *et al*, 1998; Gabrilove *et al*, 2001). Epoetin alfa has also been shown to cross the blood–brain barrier and exhibit neuroprotective effects in preclinical studies (Brines *et al*, 2000; Cerami *et al*, 2001); and clinical trials in anaemic patients with chronic renal failure suggest that correction of anaemia with epoetin alfa improves brain and cognitive function (Marsh *et al*, 1991; Pickett *et al*, 1999).

The results from a study evaluating the effects of epoetin alfa on transfusion requirements and haematopoietic response, together with a univariate analysis of the study's QoL data, have been published in detail elsewhere (Littlewood *et al*, 2001). However, univariate analysis did not take into account possible between-group differences in factors that could influence QoL outcome. Therefore, to assess more precisely the effects of epoetin alfa treatment on QoL, these data were additionally examined by *a priori*-planned multiple linear regression analysis, which controlled for disease progression and other possibly confounding variables (e.g., demographic and baseline clinical characteristics). In this study, we present the findings of the multiple linear regression analysis and explore more fully the relationship between Hb level and QoL. We also take this opportunity to describe the design and results from this study more fully than was possible in the article by Littlewood *et al* (2001).

## PATIENTS AND METHODS

### Patients and study design

The design of the study has been described previously (Littlewood *et al*, 2001). Briefly, the randomised, double-blind, placebo-controlled trial was conducted in 15 countries at 73 sites with 375 patients (intent-to-treat (ITT) population). The QoL analysis population comprised those ITT patients who had both a baseline and at least 1 follow-up QoL assessment.

To be enrolled, patients were required to have a confirmed diagnosis of solid or non-myeloid haematologic malignancy for which they may or may not have been receiving non-platinum chemotherapy, but were scheduled to receive (an additional) 12–24 weeks (3–6 cycles) of such therapy. Only patients receiving non-platinum regimens could be included in this phase III study because epoetin alfa was already licensed worldwide for use in patients receiving platinum regimens. In addition, at screening (approximately 1 week prior to randomisation to study drug), patients were required to have Hb levels ⩽10.5 g dL^−1^, or levels >10.5 g dL^−1^ but ⩽12 g dL^−1^ following a ⩾1.5-g dL^−1^ decrease in Hb per cycle or month since starting chemotherapy. Patients with acute leukaemia or myeloid malignancies were excluded, as were those with untreated iron, folate, or vitamin B_12_ deficiency. Also excluded were patients who had acute major infection or bleeding within 1 month, radiation therapy or allogeneic blood transfusion within 2 weeks, or surgery or acute major illness within 1 week prior to study entry. All patients provided written informed consent before undergoing any study-related procedures.

Patients were stratified by tumour type (solid or haematologic) and Hb level (⩽10.5 g dL^−1^, or >10.5 but ⩽12 g dL^−1^) at screening. They were randomised in a 2 : 1 ratio, balanced by using permuted blocks, to receive epoetin alfa 150–300 IU kg^−1^ three times per week or matching placebo by subcutaneous injection. (Outside of the United States, epoetin alfa is manufactured by Ortho Biologics, LLC and distributed and marketed as EPREX® or ERYPO® by Ortho Biotech and Janssen-Cilag. In the United States, PROCRIT® (epoetin alfa) is manufactured by Amgen Inc. and distributed and marketed by Ortho Biotech Products, L.P.) Study treatment (i.e., epoetin alfa or placebo) was administered for the duration of the study, a maximum of 28 weeks. Study duration included 12–24 weeks (3–6 cycles) of chemotherapy and a 4-week period when study treatment continued after the last dose of chemotherapy.

The sample size for the trial was determined in consideration of finding a significant effect for the primary efficacy endpoint (transfusions after the first 28 days on-study). Specifically, the sample size calculation was based on detecting an odds ratio of 2 between success and treatment, where success is defined as the absence of any transfusion after the end of the first 4 weeks of treatment. Given a power of 90%, a significance level of .05 (one-sided), and based on the Cochran-Mantel-Haenszel test, the sample size needed was *a priori* calculated as 120 in the placebo arm and 240 in the epoetin alfa arm, resulting in a total sample size of 360 (patients randomised to epoetin alfa and placebo in a ratio of 2 : 1).

### Evaluation of quality of life

The QoL hypothesis for this study was that administration of epoetin alfa would improve QoL relative to placebo due to treatment-induced improvements in Hb levels. Clinical efficacy endpoints included the proportion of patients transfused after the first 4 weeks of anaemia treatment (primary endpoint) and change in Hb level from baseline to last available value (secondary endpoint). Change in QoL from baseline to last available assessment was also a secondary efficacy endpoint. The effects of epoetin alfa on QoL were assessed using three patient-rated QoL instruments: subscales of the Functional Assessment of Cancer Therapy-Anaemia (FACT-An), the Cancer Linear Analogue Scale (CLAS, also known as Linear Analogue Scale Assessment, or LASA), and the Medical Outcomes Study Short Form-36 (SF-36). The FACT-An is a 47-item, cancer-specific questionnaire comprising a core 27-item general questionnaire (FACT-G Total) that measures physical, social/family, emotional, and functional well-being, and an additional 20-item anaemia questionnaire that assesses the impact of fatigue (13 items, fatigue subscale (FACT-An Fatigue subscale)) and anaemia (seven items, anaemia subscale (FACT-An Anaemia subscale)). The CLAS, another cancer-specific instrument, consists of three QoL visual analogue scales that measure energy level, ability to do daily activities, and overall QoL using a 100-mm scale. The SF-36 is a general health-assessment instrument that provides scores for eight QoL dimensions as well as summary scores for two derived measures (Physical Component Summary and Mental Component Summary (PCS and MCS, respectively)). Possible scores from the FACT-G Total scale range from 0 to 108; for the FACT-An Fatigue subscale, scores range from 0 to 52; and for the three CLAS items scores range from 0 to 100. The SF-36 PCS and MCS scales have no meaningful lower or upper bounds, but are standardised to have a mean of 50 and a standard deviation of 10 points based upon a representative sample from the United States population. The procedure for deriving the standardised PCS and MCS scores has been cross-culturally validated by the instrument developers (Bullinger *et al*, 1998). Higher scores represent better QoL in every case.

Five QoL scales (FACT-G Total, FACT-An Fatigue subscale (Cella, 1997; Demetri *et al*, 1998), CLAS : Energy, CLAS : Ability to Do Daily Activities, CLAS : Overall QoL (Cella, 1997; Glaspy *et al*, 1997; Demetri *et al*, 1998)) have demonstrated sensitivity to Hb levels and were therefore designated *a priori* as the primary QoL efficacy endpoints for analyses of the effects of epoetin alfa. The SF-36 PCS and MCS scales were additionally designated as primary QoL endpoints for analyses, and were included as general measures of QoL.

**Figure 1:**
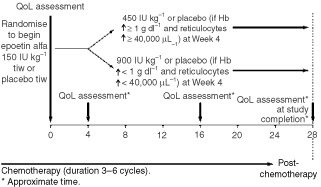
Study schema.

Patients self-administered the three QoL questionnaires as a single packet a maximum of four times during the study: (1) pretreatment (at the time of randomisation to either epoetin alfa or placebo); (2) before the start of the second on-study chemotherapy cycle for patients on a cyclic chemotherapy regimen (or approximately week 4); (3) before the start of the fifth on-study chemotherapy cycle (or approximately week 16); and (4) at study completion (within 5 days of study completion or at the time of early withdrawal) (Figure 1

). QoL questionnaires were filled out by the patients at the beginning of the relevant study visits, before any other tests, procedures, or consultations. In this way, reported QoL was masked with respect to current clinical status. No provisions were made for caregivers or other proxies to fill out the QoL questionnaires in cases where the patients were unable to do so. Each patient enrolled in the study was expected to complete the QoL assessments (where available in his or her language).

**Figure 2:**
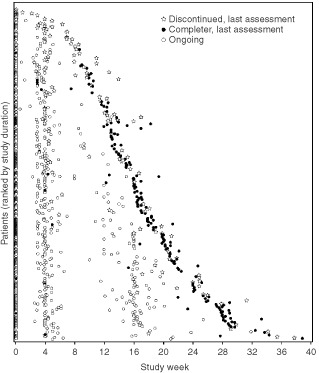
Timing of QoL assessments. Each symbol represents a QoL assessment for a patient at the specified time in the study. The three separate symbols represent the last assessment for a patient who withdrew early (star), the last assessment for a completer (closed circle), or a continuing assessment (open circle).

Even if a patient received more than six cycles of chemotherapy, the study was scheduled to end at a maximum of 28 weeks. Patients were enrolled into the study if they were expected to undergo a minimum of three and a maximum of six (additional) cycles of chemotherapy. However, chemotherapy may have been discontinued earlier as a result of disease progression or toxicity of the chemotherapeutic regimen. Therefore, some patients who did not begin a second cycle of chemotherapy, and thus did not self-administer the first follow-up QoL assessment, may still have completed the epoetin alfa study. In such cases, patients were asked to complete the QoL assessment scheduled for study completion. Because patients could be enrolled with a variable number of expected chemotherapy cycles, and because those cycles could be of varying durations, the timing of QoL assessments could not be standardised to specific days or weeks in the study. The actual timing of the QoL assessments that resulted from this study design is illustrated in Figure 2

, where the vertical axis represents patients (sorted by days in the study) and the horizontal axis represents study duration. Missing assessments were not imputed.

### Statistical methods

For all analyses, QoL change scores were calculated by subtracting the baseline score from the score obtained at the last available assessment. Changes in QoL data were examined by univariate and multiple linear regression analyses. Univariate analysis methods have been published elsewhere (Littlewood *et al*, 2001). Multiple linear regression analyses, which were planned *a priori*, were performed because previous studies indicated that disease progression has a profound effect on QoL scores at follow-up (Demetri *et al*, 1998). Therefore, including disease progression and other confounding factors in each model elucidates pure treatment effect on QoL by adjusting for the effects of these factors. These multiple linear regression analyses were performed as a validation test for the originally published univariate analyses.

For the multiple linear regression analyses, separate analyses of covariance models were estimated for each of the seven primary QoL endpoints using the SAS General Linear Model (GLM) procedure (SAS Institute, Inc., Cary, NC, USA). In all models, the QoL change score was the dependent variable. Independent variables included treatment with epoetin alfa, disease progression, treatment interaction with disease progression, and several selected demographic and clinical variables (age, sex, race, baseline Hb level, baseline neutrophil count, baseline reticulocyte count, baseline erythropoietin level, prestudy transfusion dependence, and tumour type (solid or haematologic)). Variables representing treatment with epoetin alfa and disease progression and its interaction with treatment were included in every model. Response to chemotherapy was collected at study termination or early withdrawal. A progressive disease flag was constructed from the chemotherapy response values by setting the flag to one if the response was Progressive Disease (increase in estimated tumour mass by ⩾25% or appearance of new lesion), and by setting the flag to zero otherwise. Patients who were not assessed were categorised as ‘Unknown’ (i.e., not progressive disease). All other independent variables were retained in the model if they were significant at or below the 10% level. Mean QoL change was estimated for each treatment group from the regression parameter estimates using the Least Squares Means (LSM) method (Searle *et al*, 1980). T statistics were then generated to test for differences in mean change scores between the treatment groups. All significance tests were two-sided, with the alpha level set at 0.05, adjusting for multiple comparisons using a sequentially rejective version of the Bonferroni procedure (Hochberg, 1988).

The LSM analysis was performed over the entire QoL analysis population adjusted for all covariates, including disease progression. From this analysis, the overall mean changes in QoL for the two treatment groups were estimated. However, because disease progression and its interaction with treatment were included in each multiple linear regression model, LSMs of adjusted QoL change scores could also be generalised to two separate patient populations: a population containing only patients exhibiting disease progression and another containing only patients not exhibiting disease progression (i.e., stable, partial, or complete responders to chemotherapy). It should be emphasised that the LSM analysis of patients exhibiting disease progression was not a subgroup analysis; it was an estimate resulting from the regression parameters calculated over all study patients in the entire QoL cohort. The results of such an analysis can be interpreted as predicting the treatment-related QoL benefit that would be expected for a hypothetical patient exhibiting disease progression or for a hypothetical patient not exhibiting disease progression. Of course, in practice, clinicians cannot generally predict which patients will progress. However, because disease progression has such a detrimental effect upon patient QoL, we have explicitly included that possibility in our model specification. This addresses the criticism recently made by Bottomley *et al* that most QoL papers investigating the effects of epoetin alfa do not take tumour response into account (Bottomley *et al*, 2002).

Given the underlying QoL hypothesis that administration of epoetin alfa would improve QoL relative to placebo due to treatment-induced improvements in Hb levels, Pearson correlation coefficients were calculated to assess the relationship between baseline Hb level and QoL (cross-sectional correlation), and between change in Hb level and change in score for each primary QoL endpoint (longitudinal correlations). *P* values associated with these correlation coefficients were also adjusted for multiple comparisons using the sequentially rejective Bonferroni procedure.

## RESULTS

Table 1

**Table 1:**
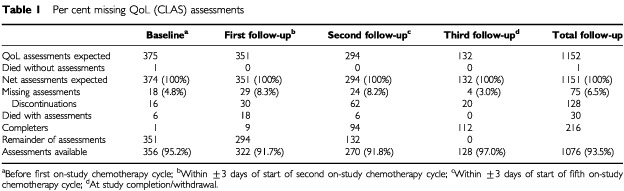
Per cent missing QoL (CLAS) assessments

presents the QoL assessments expected at baseline and follow-up based upon the study design and timing of discontinuing patients. There was a minimal amount of avoidable QoL data missing in this trial due to administrative or logistical reasons (approximately 6.5%, shown as missing CLAS data in Table 1). As seen in the last column of Table 1, 1076 of 1151 QoL assessments expected were available. In the context of clinical studies in oncology, this represents a very low rate of avoidable missing data (Bernhard *et al*, 1998).

Generally, there was a very low rate of missing QoL items (as opposed to multi-item scales), given the completion of an assessment (Table 2

**Table 2:**
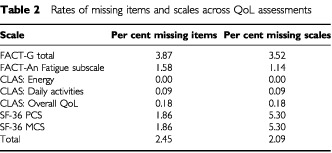
Rates of missing items and scales across QoL assessments

). Over the seven QoL scales chosen *a priori* for analysis, there was approximately a 2.45% rate of missing items. The FACT-An and the SF-36 scales can be scored in the presence of some level of missing items, but those multi-item scales do mandate a requisite number of non-missing items (usually 50% non-missing). Thus, the missing scale rates for the seven QoL scales chosen *a priori* for analysis are also presented in Table 2. As the table shows, there was a 2.09% missing scale rate for these seven scales combined. Because the SF-36 PCS and MCS are derived from the same set of items, the percent missing for these two scales is equivalent.

In addition to the data missing due to administrative or logistical reasons, 54 patients in the ITT population had no FACT or SF-36 data owing to unavailability of these questionnaires in some languages (i.e., Czech, Greek, Hungarian, Polish), and an additional 23 were missing those data for other reasons. The CLAS questionnaire was available and administered in the languages named above, as well as in Dutch, English, French, German, Italian, and Portuguese, but 39 patients had missing CLAS data for non-language-related reasons. Therefore, a total of 298 (79.5%) and 336 (89.6%) patients from the ITT population were included in the FACT and CLAS analyses, respectively. The numbers of patients from each country whose FACT or CLAS data were available for analysis are shown in Table 3

**Table 3:**
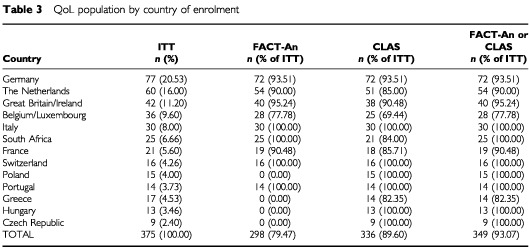
QoL population by country of enrolment

. Figure 3

**Figure 3:**
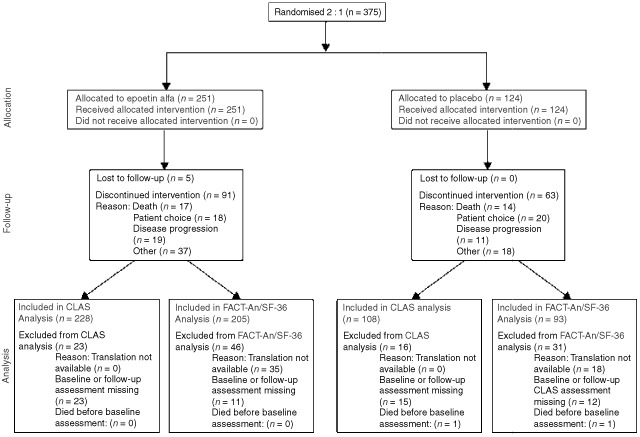
Flow diagram of the patients' progress through the phases of the trial.

is a flow diagram, recommended for use by the CONSORT statement, that displays patients' progress through the phases of the trial (Moher *et al*, 2001).

### Baseline characteristics

Baseline characteristics of the QoL population by covariate are summarised in Table 4

**Table 4:**
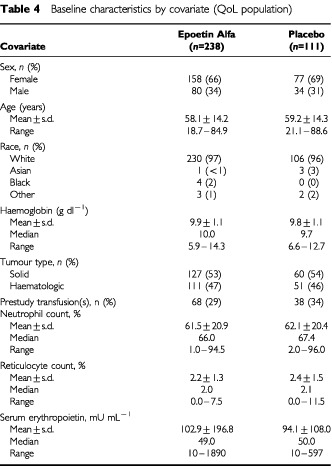
Baseline characteristics by covariate (QoL population)

. These characteristics were generally comparable between the epoetin alfa- and placebo-treated patients. Ninety-two per cent of patients in each treatment group had begun chemotherapy within 3 months of the beginning of the study (Littlewood *et al*, 2001). Eligibility for the study was limited to patients whose Hb was ⩽12 g dL^−1^ at screening; however, because some patients may have received transfusions or ceased chemotherapy treatment following screening, their Hb may have risen higher than 12 g dL^−1^ by the time their baseline measurements were assessed. These patients were included in the study and in the ITT analyses.

### Transfusion needs and haematopoietic response

Fewer epoetin alfa-treated patients than placebo-treated patients in the ITT population were transfused after day 28 (24.7% *vs* 39.5%, *P*<0.01, Wald's χ^2^ test from logistic model correcting for tumour type and Hb stratum) (Littlewood *et al*, 2001). (For this analysis, patients on study 28 days or less were counted as transfused, that is, as failures.) Patients treated with epoetin alfa had a significantly greater increase in mean (±s.d.) Hb level than did those treated with placebo (2.2±2.18 g dL^−1^ *vs* 0.5±1.79 g dL^−1^, *P*<0.01, *t*-test). (Transfusion-related changes in Hb level were included in the calculation of mean change.) This pattern was observed regardless of tumour type (solid *vs* haematologic) or Hb stratum (Littlewood *et al*, 2001).

### Changes in quality of life

The multiple linear regression models and parameter values used to generate LSMs for each of the analyses in this report are presented in Table 5

**Table 5:**
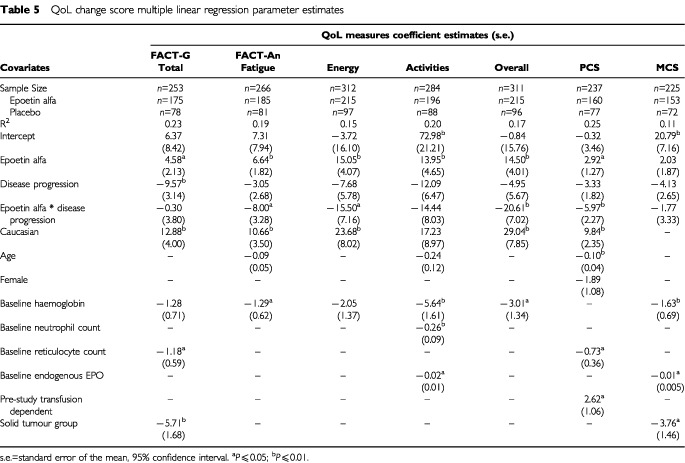
QoL change score multiple linear regression parameter estimates

. Table 6

**Table 6:**
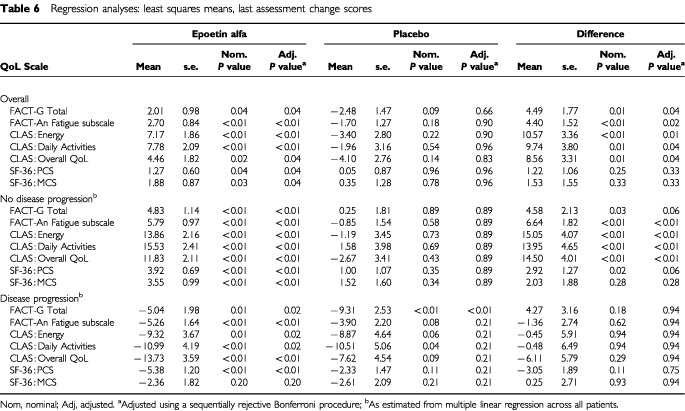
Regression analyses: least squares means, last assessment change scores

presents LSM estimates for the seven primary QoL endpoints for the overall analysis and by disease progression status. Results of both the univariate and multiple linear regression analyses of QoL change scores for the two treatment groups are displayed in Figures 4

**Figure 4:**
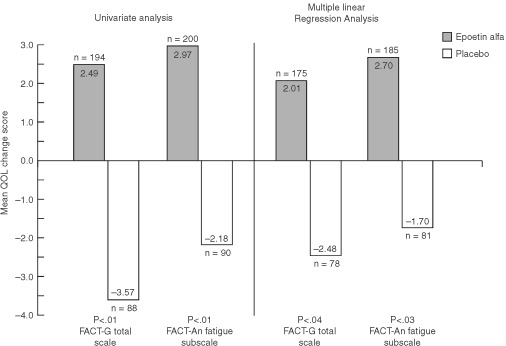
QoL mean change scores by treatment group: results of univariate (Littlewood *et al*, 2001) and multiple linear regression analyses* (FACT-G Total scale and FACT-Fatigue Subscale). *P values adjusted for multiple comparisons (sequentially rejective Bonferroni procedure).

, 5

**Figure 5:**
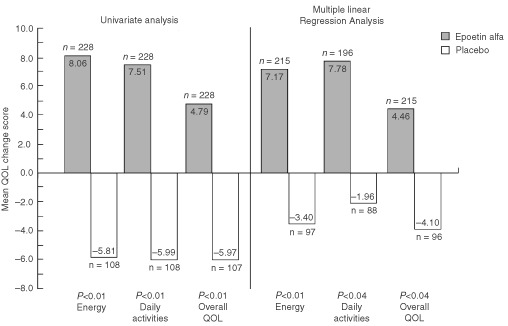
QoL mean change scores by treatment group: results of univariate (Littlewood *et al*, 2001) and multiple linear regression analyses* (CLAS scales). *P values adjusted for multiple comparisons (sequentially rejective Bonferroni procedure).

, and 6 with the previously published (Littlewood *et al*, 2001) univariate analyses QoL results included as a reference.

**Figure 6:**
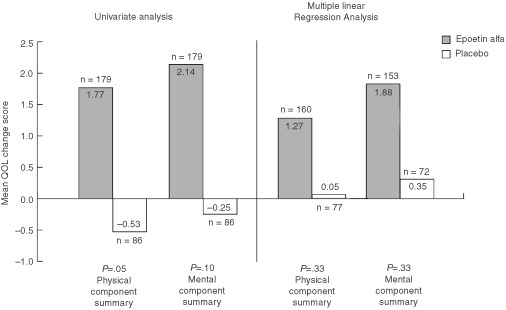
QoL mean change scores by treatment group: results of univariate (Littlewood *et al*, 2001) and multiple linear regression analyses* (SF-36 Summary scores). *P values adjusted for multiple comparisons (sequentially rejective Bonferroni procedure).

After adjusting for possible differences in demographic and clinical characteristics between the treatment groups, results of the multiple linear regression analyses confirmed the results of the univariate analyses. All within-group mean change scores for the epoetin alfa group were again positive (Figures 4,5,6

). Further, mean changes for all seven of these scores were statistically significant (range, *P*<0.01 to *P*<0.05) (Table 6). In contrast, within-group mean change scores for the placebo group were negative, except for the SF-36 MCS and PCS (Figures 4,5,6). For the overall QoL population, between-group comparisons of the LSM QoL change scores showed statistically significant differences favouring epoetin alfa for all five cancer-specific scales (range, *P*=0.01 to *P*=0.04) but not for the two SF-36 scales (Figures 4,5,6, Table 6). However, the two SF-36 scales showed no evidence of deterioration in overall physical or mental QoL for patients treated with epoetin alfa.

As estimated by the multiple linear regression model, a hypothetical patient treated with epoetin alfa and exhibiting no disease progression can expect significant benefit as measured by the FACT-An Fatigue subscale and all three CLAS scales. However, a hypothetical patient exhibiting disease progression (in this study, approximately 28% of the QoL population experienced disease progression) could expect no benefit in these measures as based upon the multiple linear regression results (Table 6). These results can be seen as validating the sensitivity of the QoL instruments used in this study. One would expect that in a general population of cancer patients (i.e., a population containing both responders and non-responders to cancer treatment), a significant degradation in patient QoL would generally be associated with significant disease progression, regardless of cancer-related anaemia treatment.

### Correlation between haemoglobin and quality of life

Correlation analyses were performed to examine the relationship between Hb level and QoL (Table 7

**Table 7:**
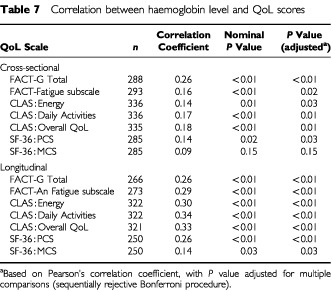
Correlation between haemoglobin level and QoL scores

). Positive cross-sectional correlations were found for every scale except the SF-36 MCS scale. Slightly stronger longitudinal correlations were found using all seven primary QoL scales; all *P* values were significant (range, *P*<0.01 to *P*=0.03). Figure 7

**Figure 7:**
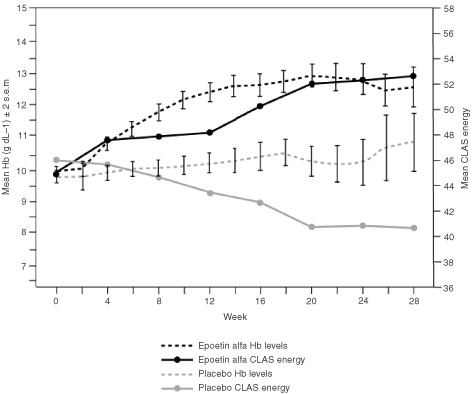
Visual graphic demonstrating the relationship between Hb and QoL over time by showing biweekly Hb levels and CLAS Energy levels from univariate analysis (Littlewood *et al*, 2001).

demonstrates the relationship between Hb and QoL over time by showing biweekly Hb levels (Littlewood *et al*, 2001) and monthly CLAS Energy levels. While the QoL assessments were not designed in this study to yield monthly mean scores and no analysis of monthly mean scores was conducted or reported in this study, this graph does help to visualise the longitudinal relationship between Hb and QoL. The monthly CLAS Energy levels shown in this graph were derived using 28-day windows for months, with the last observation carried forward for missing values and dropouts. It is possible that the decline in QoL seen in the placebo arm, despite relatively stable but low Hb levels, may suggest that long-term anaemia has a cumulative effect on QoL, especially during chemotherapy. The significant correlations seen in Table 7, and QoL response to Hb shown in Figure 7, suggest that the administration of epoetin alfa in this patient population improves health-related QoL, which is associated with treatment-induced improvements in Hb levels.

### Safety

Information regarding adverse events has been reported in detail elsewhere (Littlewood *et al*, 2001). Epoetin alfa was well tolerated, and the overall incidence of adverse events and the incidence of individual adverse events were generally similar between the two treatment groups.

## DISCUSSION

Assessment of QoL has now become a standard, and in some cases mandatory, endpoint in oncology clinical trials and studies. This increased focus on QoL as a therapeutic outcome is being driven by advances made in cancer treatment and management over the last few decades. Earlier detection of malignancies and widespread availability of cancer screening programmes have increased the possibility of successful treatment and recovery, although often at the cost of functional ability and physical, emotional/mental, or social well-being. Also, more intense treatment strategies are being employed with the potential for greater toxicity and associated negative impact on QoL. Determination of the QoL outcome for any potential therapy, whether cancer-specific or supportive, is therefore essential for evaluating comparative treatments as well as making decisions about future treatment or palliative care (Cella, 1997). The primary therapeutic endpoints of concern to the clinical scientist may be very different from those of patients and, consequently, the value placed on gaining relief from symptoms may differ also. Thus, to make informed decisions about the benefits of supportive treatments that have cost implications, both doctors and their patients need the information gained from comprehensive QoL evaluation, not merely clinical or economic evaluation.

The FACT-G Total, FACT-An Fatigue subscale, CLAS, and SF-36: PCS and MCS scales were chosen as primary QoL endpoints to assess the effects of epoetin alfa on QoL in the present study. The FACT-G Total, FACT-An Fatigue subscale, and CLAS were considered especially appropriate for this purpose as they are cancer-specific, Hb-sensitive, and easily completed by patients. The CLAS, in addition, had been used frequently in previous trials of epoetin alfa and, at the time this study was initiated, was more widely available than the FACT scales in the languages of the participating countries (10 *vs* six languages). Also, its use could facilitate comparisons between the results of this study and previous trials. The SF-36 is not cancer-specific but was included as a general measure to ensure that there were no unexpected adverse consequences of epoetin alfa therapy affecting overall QoL.

To clarify the effect of epoetin alfa on QoL, beyond that originally reported based upon univariate analyses (Littlewood *et al*, 2001), a multiple linear regression analysis was performed that took into account tumour progression as well as many other possibly confounding variables (e.g., age, sex, baseline Hb). As in the univariate analysis, the *P* values were adjusted to account for multiple comparisons. The results of this analysis confirmed the advantage of epoetin alfa over placebo, as demonstrated in the univariate analysis, with significant differences again noted for all cancer-specific scales. While the generality of the two SF-36 summary scales reduced their ability to discriminate between groups in this analysis, the positive (though non-significant) SF-36 changes favouring epoetin alfa strongly suggest that there were no general trends in unforeseen detrimental effects on QoL from epoetin alfa treatment using an instrument that casts a wide net for functional and well-being domains. In fact, it should be noted that the within-group analysis shows a positive and statistically significant change in the two SF-36 scales for the epoetin alfa group, but no corresponding significant change for the placebo group.

While only 2–3% of the patients included in the current report were non-Caucasian, race was included as a covariate because it has historically been a strong predictor of QoL and is customarily included in multiple linear regression analyses of treatment effect on QoL. The variable's highly significant parameter estimates, despite the low number of non-Caucasian patients included in the study, indicates that race may be an important predictor of patient QoL. For this reason, and to maintain consistency with the main body of QoL research, we decided to retain race in our regressions.

A possible limitation of this analysis is the focus upon the QoL change from baseline to the last available assessment. Analyses incorporating change scores derived in this manner may be affected by bias across treatment arms. For example, patients in one treatment arm may stop early due to anaemia (and thus a low QoL) whereas in the other arm patients may stop due to other reasons (and perhaps their last assessment will be a normal QoL). We chose to define the QoL change score in this manner to remain consistent with the results reported in Littlewood *et al* (2001) for both the QoL and the other clinical measures. However, a separate sensitivity analysis was conducted that incorporated the longitudinal nature of the Littlewood *et al* (2001) data. This sensitivity analysis included all available data from each patient and also adjusted for censored assessments (Fairclough and Gagnon, 2000). Compared with the baseline-to-last assessment analysis described in this study, the sensitivity analysis found that the between-group differences remained similar between the two analytic techniques.

While the results reported here are statistically significant, the question remains whether they are clinically relevant. By examining Figures 4 and 5, one can see a between-group difference of 4 to 5 points in FACT-G Total and FACT-An Fatigue subscale change scores and a between-group difference of 8.5–10.6 points for the three CLAS scales. The question of the clinical relevance of these changes can be partially answered by the results of two recent analyses. An analysis based on the Littlewood *et al* study compared the FACT-An and CLAS score changes of improved and stable patients; differences in corresponding QoL scores were considered to be the minimally important differences (MIDs) (Patrick *et al*, 2000). For the FACT-G Total and Fact-An Fatigue subscale, the MIDs were 2.25 and 4.23, respectively. For the CLAS scales, the MIDs ranged from 8.98 to 10.26. The between-group differences observed from the multivariate analyses exceeded the MIDs for all scales except the CLAS Overall QoL. A separate analysis compared the FACT-An scores from the Littlewood *et al* (2001) trial with scores from a demographically representative United States sample (Nortier *et al*, 2001). The analysis found that the between-group QoL advantage seen in epoetin alfa-treated patients represented 95% and 51% of the deficit from normative scores seen at baseline for the FACT-G Total and FACT-An Fatigue subscale, respectively. Based on the conclusions of these analyses, the results for the cancer-specific scales seen in the clinical trial appear not only to be statistically significant, but also clinically relevant.

Due to space limitations and, often, trial design, QoL results (if they are published at all) are usually presented in a fashion similar to that used for physiologic endpoints (i.e., a simple statistical comparison of ITT groups). The current study is given to rigorous analysis including multiple linear regression on over a dozen covariates, consideration of patterns of missing data, and further interpretation of the robustness of the results based upon these analyses. Further, the finding of a positive within-group epoetin alfa treatment effect on cancer-specific QoL is corroborated by several randomised placebo-controlled and open-label studies (Abels, 1992; Glaspy *et al*, 1997; Demetri *et al*, 1998; Gabrilove *et al*, 2001). In the earliest of these studies (Abels, 1992), QoL was assessed in a series of placebo-controlled trials conducted in three different patient populations: patients receiving no chemotherapy, patients receiving non-cisplatin-containing chemotherapy, and patients receiving cisplatin-containing chemotherapy. QoL was evaluated by means of visual analogue scales that measured energy level, ability to do daily activities, and overall QoL (scales equivalent to the CLAS scales used in the present study). Comparison of QoL scores for the total epoetin alfa population of the three trials with those for the total placebo population revealed significantly (*P*<0.05) greater improvement in all three QoL parameters in patients who responded to epoetin alfa (i.e., had a haematocrit increase ⩾6%) *vs* patients given placebo. From this, the investigators concluded that epoetin alfa does improve functional capacity in anaemic cancer patients when there is an increase in haematocrit.

In the three separate large, open-label, non-placebo-controlled, community-based surveillance studies with a combined enrolment of over 7000 patients, large positive changes were noted in Hb levels and QoL of patients treated with epoetin alfa (Glaspy *et al*, 1997; Demetri *et al*, 1998; Gabrilove *et al*, 2001). Transfusion requirements also decreased steadily and dramatically throughout the course of all three studies. These changes were noted even after accounting for disease progression in two of the studies (Glaspy *et al*, 1997; Demetri *et al*, 1998). The relationship between disease progression and QoL changes was noted to be similar to the results we present here, confirming that patients with disease progression have a much larger deterioration in QoL than those without, but also concluding that anaemia made a large and independent contribution to QoL in all subgroups of patients. Of note, patients with disease progression having an improvement in Hb of greater than 2 g dL^−1^ were able to maintain their QoL at or near baseline levels while those with both disease progression and no improvement in anaemia experienced deterioration in QoL. Finally, there were significant correlations noted in these studies between Hb change and change in QoL (Glaspy *et al*, 1997; Demetri *et al*, 1998; Gabrilove *et al*, 2001), even after accounting for the effects of disease progression (Glaspy *et al*, 1997; Demetri *et al*, 1998).

Although both the Abels study and the community-based studies support the main finding of the present study, each study had limitations, the most obvious being that the community-based studies were not placebo-controlled. It is, however, an important finding that several large, community-based studies that more closely represent the actual clinical environment corroborate the results seen in the present randomised, placebo-controlled study. The Abels study used visual analogue scales to measure changes in symptoms and QoL, a method that is recognised in pain assessment and other symptom assessment and has sensitivity in cancer-symptom assessment. Since then, new instruments such as the FACT have been developed to augment measurements such as linear analogue scales; and studies using both measures now report positive changes in both instruments with anaemia correction (Glaspy *et al*, 1997; Demetri *et al*, 1998). In addition, the Abels study reported differences in results between patients who received placebo and patients who responded to epoetin alfa treatment rather than reporting results in all patients who received epoetin alfa.

The finding in the present study of an important role for Hb levels in increasing and maintaining QoL also is in agreement with the findings of two other studies that investigated the impact of anaemia and fatigue on cancer patients (Cella, 1997; Langer *et al*, 1997). In one study (Cella, 1997), assessment of QoL based on the FACT-An showed that patients with Hb levels >12 g dL^−1^ had significantly less fatigue, fewer non-fatigue anaemia symptoms, better physical and functional well-being, and higher overall QoL than those with an Hb level ⩽12 g dL^−1^ (*P*⩽0.01 for all). The second study (Langer *et al*, 1997), using an index based on FACT-Lung subscales, showed a significant correlation (*r*=0.38, *P*⩽0.02), independent of tumour response, between worsening anaemia and diminishing QoL in patients with non-small-cell lung cancer by their fourth cycle of chemotherapy. Together with data presented here, these studies provide objective evidence that increasing Hb levels in anaemic cancer patients can positively influence their QoL (Cella, 1997; Glaspy *et al*, 1997; Langer *et al*, 1997; Demetri *et al*, 1998; Gabrilove *et al*, 2001).

## CONCLUSIONS

In summary, the results of univariate analysis of change scores for cancer-specific scales that evaluated QoL in this randomised study in anaemic patients receiving non-platinum-based chemotherapy were confirmed by multiple linear regression analysis, which adjusted for possible differences in demographic and clinical characteristics between the treatment groups as well as tumour response. Correlation analysis revealed a significant positive relationship between change in Hb level and change in all primary cancer-specific QoL endpoints evaluated. These findings indicate that epoetin alfa can improve QoL in anaemic cancer patients undergoing chemotherapy, and this change is associated with increasing Hb levels. Moreover, the results demonstrate that epoetin alfa treatment and disease progression are independent contributors to changes in QoL.

In the increasingly cost-contained and cost-conscious clinical environments in which many European clinicians work, there might well be reluctance to prescribe a relatively expensive product. The importance of successfully treating chemotherapy-induced anaemia by raising Hb levels with epoetin alfa and the concomitant benefits that this brings for patients' functioning and well-being are now clear. Providers are therefore faced with a situation similar to that experienced with the advent of the 5HT3 antagonists. Although nausea and vomiting had a severely deleterious effect on the quality of patients' lives and these symptoms were always ranked at the top of lists charting patients' concerns, some clinicians were either reluctant or unable to prescribe the most efficacious treatments on the grounds of cost. This was eventually seen as a weak argument, as uncontrolled symptoms that detract from patients' QoL can jeopardise completion of treatment and may have many hidden or unmeasured costs. Consequently, 5HT3 antagonists are now regarded as a standard part of the management of patients undergoing chemotherapy and this has transformed the lives of many patients, making chemotherapy much more tolerable.

Now that nausea and vomiting in chemotherapy have been significantly alleviated, fatigue due to the chemotherapy-induced anaemia has emerged as a major and distressing problem for patients (Curt *et al*, 2000). It is usually poorly understood, often managed inadequately, and its impact on patients is generally underestimated. We hope that this study will help to raise awareness that epoetin alfa significantly improves Hb levels and reduces the fatigue that so impairs the QoL of anaemic cancer patients.

## Appendix

The following investigators participated in this study: *Belgium*: Yves Beguin, MD, CHU Sart-Tilman, Liege; Rene Brys, MD, H. Hart Klinick, Ecklo; Gilbert De Wasch, MD, Henri Serruys Hospital, Oostende; Mario Antonio Dicato, MD, Centre Hospitalier de Luxembourg, Luxembourg; Raymond Mathys, MD, A.Z. Middelheim, Antwerpen; M. Symann, MD, Oncologie Médicale, Cliniques St Luc, Brussels; Achiel Van Hoof, MD, Algemeen Ziekenhuis St Jan, Brugge; and Ignace Vergote, MD, U.Z. Gasthuisberg, Leuven. *Czech Republic*: Otakar Bednarik, MD, Masaryk Memorial Cancer Institute, Brno; Michael Frank, MD, Department of Radiotherapy, Faculty Hospital, Plzen; and Milada Zemanova, MD, Veobecná Fakultní Nemocnice UK, Praha 2. *France*: Pierre-Etienne Cailleux, MD, Service de Radiotherapie/Chimiotherapie, Tours; Herve Cure, MD, Centre Regional de Lutte, Contre le Cancer Jean Perrin, Clermont-Ferrand; Marine Divine, MD, Hospital Henri Mondor, Creteil; Jean-Paul Guastalla, MD, Centre Regional de Lutte, Contre le Cancer Leon Berard, Lyon; M. Faress Husseini, MD, Hospital Pasteur, Hospitaux Civils Colmar, Colmar; and Hervé Lacroix, MD, CHU-Hospital Laennec, Service d'Oncologie Generale, Nantes. *Germany*: Gerhard Adam, MD, Asklepios Klinik Triberg, Triberg; Konstantin Akrivakis, MD, Universitätsklinikum Charité, Berlin; Carsten Bokemeyer, MD, Medizinische Universitätsklinik Abt. II, Eberhard-Karls Universität, Tübingen; Lothar Böning, MD, Onkologische Praxisgemeinschaft, München; Mathias Freund, MD, Abteilung Hämatologic/Onkologic, Klinik und Poliklinik für Innere Medizin, Rostock; Hans-Jürgen Hurtz, MD, Onkologische Gemeinschaftspraxis, Halle; Hartmut Kirchner, MD, Siloa Hospital, Clinic for Hematology and Oncology, Hannover; Erhard Kurschel, MD, Gemeinschaftspraxis, Oberhausen; Wolf-Dieter Ludwig, MD, Virchow-Klinikum, Robert-Rössle-Klinik, Berlin; Norbert Marschner, MD, Praxis in der Klinik für Tumorbiologie, Freiburg; Karl Ulrich Petry, MD, Frauenklinik der MHH, Hannover; Uwe Reinhardt, MD, Klinikum Bayreuth, Abteilung Onkologie, Bayreuth; Peter Reitzig, MD, Humaine Klinik, Dresden; and Wolfgang Schuette, MD, City Hospital Martha-Maria, Hämatologie, Halle. *Greece*: Vassilis Georgoulias, MD, University Hospital of Heraklion, Department of Clinical Oncology, Heraklion, Crete; Gerasimos Pangalis, MD, Laikon General Hospital, Athens; Nicholas Pavlidis, MD, University Hospital of Ioannina, Ioannina; and Dimostenis-Vasilios Skarlos, MD, Agii Anargiri Cancer Hospital, Athens. *Hungary*: Tamás Pintér, MD, Petz Aladár County Hospital, Gyõr, Department Oncoradiology, Gyõr; Gyula Szegedi, MD, Debrecen University Medical School, Third Department for Internal Medicine, Debrecen; and Gyula Varga, MD, Albert Szent-Gyocrgyi Med. University Szeged, Second Department of Internal Medicine, Szeged. *Ireland*: John Rory O'Donnell, MD, Beaumont Hospital, Dublin.* Italy*: Emilio Bajetta, MD, Instituto Nazionale Per Lo Studio E La Cura dei Tumori, Milan; Agostino Cortelezzi, MD, Università Degli Studi di Milano, Ospedale Policlinico, Cattedra Di Ematologia, Milan; and Gabriella Gorzegno, MD, Osperdale S. Luigi, Istituto di Clinica Medica Generale, Orbassano (Torino). *The Netherlands*: Franciscus L.G. Erdkamp, MD, Maaslandziekenhuis, Sittard; HJ Keizer, MD, Academisch Ziekenhuis Leiden, Leiden; J.J. Mol, MD, Ziekenenhuis Rijnstate, Arnhem; Johan W.R. Nortier, MD, Diakonessenhuis, Utrecht; Ron C. Rietbrock, MD, Academisch Medisch Centrum, Amsterdam; C.J. Rodenburg, MD, Algemeen Christelijk Ziekenhuis Eemland, Lokatie De Lichtenberg, Amersfoort; M.R. Schaafsma, MD, Medisch Spectrum Twente, Enschede; L.H. Siegenbeek van Heukclom, MD, Medisch Centrum Alkmaar, Alkmaar; C. Van der Heul, MD, Sint Elisabeth Ziekenhuis, Tilburg; Marinus Van Marwijk Kooy, MD, St Sophia Ziekenhuis, Zwolle; Gerard Vreugdenhil, MD, Sint Joseph Ziekenhuis, Veldhoven; and Jacques Wils, MD, Laurentius Hospital, Roermond. *Poland*: Jerzy Holowiecki, MD, Department of Haematology, Silesian School of Medicine, Katowice; Marek Pawlicki, MD, Maria Skiodowska-Curie Memorial Cancer Center, Division in Cracow, Kraków; and Piotr Siedlecki, MD, Maria Skiodowska-Curie Memorial Cancer Center, Warsaw, Warszawa. *Portugal*: Cândida Azevedo, MD, Instituto Português de Oncologia, Porto; Ricardo Marques da Costa, MD, Hospital Dristrital de Leiria, Leiria; and Joaquim Gouveia, MD, Hospital de Santo António dos Capuchos, Lisboa. *South Africa*: Dayle Hacking, MD, Durban Oncology Center, Westridge, Durban; Johann Raats, MD, 110 Delmar Medical Center, Capetown; and Bernardo Rapoport, MD, The Medical Oncology Center of Rosebank, Johannesburg. *Switzerland*: Matti S. Aapro, MD, Centre Anticancéreux, Genolier; Richard Herrmann, MD, Kantonspital Basel, Basel; J.M. Lüthi, MD, Regionalspital Thun, Thun; and Kaspar Rhyner, MD, Kantonspital Glarus, Glarus. *United Kingdom*: Peter Jeffrey Barrett-Lee, MD, Department of Oncology, Velindre Hospital, Cardiff; David Fairlamb, MD, New Cross Hospital, Ceansly Center, Wolverhampton; Riaz Jan-Mohamed, MD, Hillingdon Hospital, Uxbridge; Timothy James Littlewood, MD, John Radcliffe Hospital, Oxford; Graham Smith, MD, Royal United Hospital, Bath; and John Sweetenham, MD, Southampton General Hospital, CRC Oncology Unit, Southampton.
